# Estimation of the Costs of Invasive Cervical Cancer Treatment in Brazil: A Micro-Costing Study

**DOI:** 10.1055/s-0039-1692412

**Published:** 2019-06

**Authors:** Candice Lima Santos, Ariani Impieri Souza, José Natal Figueiroa, Suely Arruda Vidal

**Affiliations:** 1 Department of Oncology, Instituto de Medicina Integral Prof. Fernando Figueira, Recife, Brazil; 2 Medical School, Faculdade Pernambucana de Saúde, Recife, PE, Brazil; 3 Department of Research, Instituto de Medicina Integral Prof. Fernando Figueira, Recife, Brazil

**Keywords:** costs and cost analysis, cost of illness, cost measures, cervical cancer, HPV, human papillomavirus, custos e análise de custos, custo da doença, medidas de custos, câncer cervical, HPV, vírus do papilomavírus humano

## Abstract

**Objective** The main objective of the present study was to estimate the annual treatment costs of invasive cervical cancer (ICC) per patient at an oncology center in Brazil from a societal perspective by considering direct medical, direct nonmedical, and indirect costs.

**Methods** A cost analysis descriptive study, in which direct medical, direct nonmedical, and indirect costs were collected using a microcosting approach, was conducted between May 2014 and July 2016 from a societal perspective. The study population consisted of women diagnosed with ICC admitted to a tertiary hospital in Recife, state of Pernambuco, Brazil. The annual cost per patient was estimated in terms of the value of American Dollars (US$) in 2016.

**Results** From a societal perspective, the annual ICC treatment cost per patient was US$ 2,219.73. Direct medical costs were responsible for 81.2% of the total value, of which radiotherapy and outpatient chemotherapy had the largest share. Under the base-case assumption, the estimated cost to the national budget of a year of ICC treatment in the Brazilian population was US$ 25,954,195.04.

**Conclusion** We found a high economic impact of health care systems treating ICC in a poor region of Brazil. These estimates could be applicable to further evaluations of the cost-effectiveness of preventing and treating ICC.

## Abstract (pt)

**Resumo:**

**Objetivo** O objetivo principal do presente estudo foi estimar os custos anuais por paciente do tratamento do câncer do colo do útero (CCU) invasivo em um centro de oncologia no Brasil, sob a perspectiva da sociedade, considerando os custos diretos médicos, diretos não médicos e indiretos.

**Métodos** Foi realizado um estudo descritivo de análise de custos, no qual os custos médicos diretos, não médicos diretos e indiretos foram coletados por meio de uma abordagem de microcustos, realizado entre maio de 2014 e julho de 2016 sob a perspectiva da sociedade. A população do estudo foi composta por mulheres diagnosticadas com CCU invasivo internadas em um hospital terciário em Recife, PE, Brasil. O custo anual por paciente foi estimado em termos de dólares americanos (US$) para o ano de 2016.

**Resultados** O custo anual do tratamento do CCU invasivo sob a perspectiva da sociedade foi de US$ 2.219,73 por paciente. Os custos médicos diretos foram responsáveis por 81,2% do valor total, dos quais a radioterapia e a quimioterapia ambulatorial tiveram a maior participação. Sob o pressuposto do caso base, o custo estimado para o orçamento nacional de um ano de tratamento do CCU invasivo na população brasileira foi de US$ 25.954.195,04.

**Conclusão** Foi encontrado um alto impacto econômico dos sistemas de saúde para o tratamento do CCU invasivo em uma região pobre do Brasil. Essas estimativas poderão ser aplicáveis em avaliações adicionais do custo-efetividade da prevenção e tratamento do CCU.

## Introduction

Cervical cancer is the 4^th^ most common malignancy in the world.^1^ Unfortunately, in less developed countries, cervical neoplasia is still the 2^nd^ most common type of cancer in women.^2^

In Brazil, invasive cervical cancer (ICC) is the 3^rd^ most common cancer in the female population, and, in 2018, there were an estimated 16,370 new cases (15.43 per 100,000 women). Recife, a city located in the northeast region of Brazil, has one of the highest incidences of ICC (20.52 per 100,000 women) in the country.^3^

Cervical cancer screening and the treatment of precancerous lesions have successfully reduced the incidence and mortality rates of ICC in many high-income countries, but in low- and middle-income countries, infrastructure weaknesses and financing difficulties for this strategy have limited the control of cervical cancer.^4^ In addition, vaccination against the *human papillomavirus* (HPV), the etiologic agent for ICC, has been available at the Brazilian Public Health System (SUS, in the Portuguese acronym) since 2014.^5^ ^6^ Unfortunately, the benefits of HPV vaccination might not occur for many years.

The rising incidence of cancer in the world was accompanied by a huge economic cost, estimated ∼ US$ 1.16 trillion in 2010.^7^

Costing can take considerable time, and analysts need to judge how accurate cost estimates need to be conducted for a given study.^8^ There are many methods for costing patient care, and they can be separated into two main categories: “top-down” and “bottom-up” costing. Top-down costing starts with the total expenditures and then divides these by a measure of total output (e.g., radiotherapy or chemotherapy costing). Bottom-up, or microcosting, consists of identifying and costing the resources used by a specific patient, calculating the frequency of utilization of individual resources, multiplying by the cost of each unit, and then summing to achieve an overall cost.^9^

The standard treatment strategy for locally advanced ICC is concomitant chemoradiation. Despite this treatment, many patients will relapse, and some women also have metastatic disease at diagnosis and are treated with palliative chemotherapy. Recently, the addition of a molecular therapy (bevacizumab) to palliative chemotherapy in patients with recurrent, persistent, or metastatic ICC was associated with an improvement of 3.7 months in overall survival.^10^ However, two studies demonstrated that adding bevacizumab to chemotherapy would not be cost-effective in the United States if the current drug price and dose were maintained.^11^ ^12^

There are few Brazilian cost studies related to the treatment of cervical cancer, and none using the microcosting methodology. We developed this study in a tertiary hospital in Recife, state of Pernambuco, Brazil, to obtain accurate data to assist decision-makers in public policies.

## Methods

A descriptive study was conducted between May 2014 and July 2016 to determine the cost of ICC treatment for women at a public hospital (Instituto de Medicina Integral Prof. Fernando Figueira [IMIP, in the Portuguese acronym]) responsible for 27% of cancer care in Recife,^13^ a city with 1,537,704 inhabitants, of which 409,798 are women between 25 and 59 years old,^14^ with a monthly per capita income of US$ 270.81.^15^ The inclusion criteria were ICC patients with a confirmed histopathological diagnosis treated exclusively in this hospital.

The main objective was to obtain the annual per patient cost of ICC treatment at this hospital from a societal perspective by considering direct medical, direct nonmedical, and indirect costs.

Direct medical costs items included in the present analysis were surgery, chemotherapy, radiotherapy, inpatient care, laboratory and radiological tests. Direct nonmedical costs were transportation, food, and accommodation.

Direct medical costs were collected using two different approaches: top-down and bottom-up.

First, the top-down approach was used to determine the direct medical costs from the perspective of the payer (in this case, the SUS). These costs were related to the enrolled patients during the study period. Information was extracted from medical records, such as image studies performed (computed tomography [CT] scans, magnetic resonance imaging [MRI], and positron emission tomography [PET]), laboratorial tests performed, length of hospitalization, chemotherapy, and radiotherapy. Hospital and medical bills associated with each patient registry number were retrieved from the Medical Billing Department.

Second, the bottom-up approach was performed to determine the direct medical costs from the perspective of the institution (the perspective of the IMIP). This costing was collected jointly with clinical pathways to reduce missing data. Administrative files were analyzed to obtain cost data for each patient in the study period. All of the medical procedures, medications, and supplies were priced based on the financial control obtained from the purchasing sector at the institution. We calculated the frequency of utilization of each item and multiplied it by the price of the unit. Information on health professional wages in the human resources department, as well as additional hospital expenses, such as the fractions of cleaning, water, and electrical bills, were also covered.

The costs of surgical treatment using the bottom-up method could not be ascertained as a result of institutional policies during the study period.

Furthermore, interviews were conducted with the patients by the investigators to retrieve information about expenses incurred due to medical care before the hospital admission, such as medical appointments, exams, or medications.

Direct nonmedical costs were extracted from questionnaires applied to the patients, which asked about their (and their companion's) two-way transportation costs (from their home to the hospital and back), food costs, and costs of accommodations while waiting for medical procedures or appointments.

Indirect costs were obtained using two different methods. First, the Human Capital approach, as recommended by the Brazilian guidelines,^16^ obtained the number of nonworking days caused by ICC and multiplied this result by the *per capita* income. In parallel, we used the information obtained in the questionnaire regarding informal and formal jobs, social welfare, and wages. We believe this kind of approach could represent the real productivity losses of these subjects, and we can compare this result with that of the Human Capital approach.

The treatment decisions are based on the International Federation of Gynecology and Obstetrics (FIGO) stage^17^ and on the Eastern Cooperative Oncology Group (ECOG) Performance Status (PS) scores.^18^ Patients in FIGO stage IA or IB1 are referred for a modified radical hysterectomy with pelvic lymphadenectomy. Those in FIGO stage IB2 up to IVA are recommended for primary chemoradiation with weekly cisplatin during radiotherapy. For first-line treatment of metastatic disease, we indicate the use of cisplatin or carboplatin-based chemotherapy, since bevacizumab was not available in the SUS in 2016. Single agent second- and third-line chemotherapies are offered for ECOG PS 0–2 and some PS 3 patients (those who recovered with the best supportive care). Local recurrences are considered for resection. Palliative radiotherapy is offered mainly in cases of hemorrhage, pain, or epidural spinal cord compression. Patients with ECOG PS 4 are referred to palliative care.

The final cost of the present study is presented from a societal perspective. All of the costs were adjusted to the year 2016. The monetary unit used was American Dollars (US$) converted from Brazilian Real (R$) at the exchange rate of US$1 = R$3.22.^19^ In interest of global comparison, the final cost was also converted to 2016 International Dollars (I$) using purchasing power parities (PPP) from the World Bank consumer prices, converted from R$ at the exchange rate of I$1 = R$1.99.^20^ Costs incurred in 2014 and in 2015 were inflated as recommended by the Brazilian guidelines.^16^ We have used the General Market Price Index as one of the inflation indexes accepted by the Brazilian Central Bank.^21^ ^22^ ^23^ ^24^

Because future costs were not evaluated in the present analysis, we did not apply a discount rate.

Demographic parameters were analyzed with Epi Info version 3.5.3 (Centers for Disease Control and Prevention, Atlanta, GA, USA) and described in terms of means, medians, standard deviations (SDs), and interquartile ranges (IQRs). The total ICC treatment costs were obtained by adding direct medical, direct nonmedical, and indirect costs. Monetary data were processed and analyzed using Microsoft Excel for Mac version 15.30 (Microsoft Corporation, Redmond, WA, USA).

To test the differences in the mean cost between the IMIP and SUS expenses, we used the paired *t*-test in the STATA software, version 12.1 SE (StataCorp LLC, College Station, TX, USA).

Under the base case assumption, specific scenarios were explored. First, since management and reimbursements are comparable between public institutions in Brazil, and 75% of our population is assisted exclusively by the SUS,^25^ we have decided to apply the annual per patient cost results found in the present study to the Brazilian population, based on the incidence data provided by the National Cancer Institute,^3^ to estimate ICC treatment costs for Brazil.

The present project was approved by the Ethics Committee at the IMIP (document number 4026–14), and the patients agreed to provide written informed consent.

## Results

A total of 140 patients were assessed for eligibility. There were 6 exclusions due to unconfirmed diagnosis, including ovarian cancer (*n* = 1), endometrial cancer (*n* = 2), and noninvasive cervical cancer (*n* = 3), resulting in 134 patients for analysis.

The median age was 49.8 years old, with a range from 20 to 81 years old. Most of the patients (55.2%) reported having up to 3 sexual partners, the mean parity number was 4.5 children per woman, the mean schooling was 5.1 years, and 29.9% of the patients had never been to school. Out of the total sample, 38.1% of the patients were without a partner at the time of diagnosis, 56.0% were not involved in cervical cancer screening programs, and 40.3% lived < 60 km away from the IMIP Hospital. The predominant histological type was squamous cell carcinoma (81.3%), and most of the patients had advanced disease at the time of the diagnosis, with only 7.5% at FIGO Stage I. The median follow-up was of 19.4 months, and, at the time of follow-up, 83 (62%) patients had recurred, and 45 (33.6%) had died as a consequence of ICC. Most of the women were housewives (38.1%) or outside domestic workers (22.4%). The monthly mean family income was US$ 359.80. (Table 1)

**Table 1:** Sociodemographic and clinical characteristics of the women treated for invasive cervical cancer at the Instituto de Medicina Integral Prof. Fernando Figueira

Characteristics	*n* (%)
**Age group (years old)**	
20–40	36 (26.9)
41–60	73 (54.5)
61–81	25 (18.7)
**Mean (±SD)**	49.8 (±12.8)
**FIGO staging at diagnosis**	
Stage I	10 (7.5)
Stage II	53 (39.6)
Stage III	46 (34.3)
Stage IV	25 (18.7)
**Occupation**	
Housewife	51 (38.1)
Domestic worker	30 (22.4)
Merchant	14 (10.4)
Farmer	13 (9.7)
Government employee	11 (8.2)
Private employee	8 (6.0)
Retired	7 (5.2)
**Marital status**	
Married/Stable union	83 (61.9)
Single/Divorced/Widowed	51 (38.1)
**Schooling years**	
0–3	50 (37.3)
4–8	52 (38.8)
> 8	32 (23.9)
** Mean (±SD)**	5.09 (±4.43)
**Histological type**	
Squamous cell carcinoma	109 (81.3)
Adenocarcinoma	17 (1.7)
Undifferentiated carcinoma	7 (5.2)
Undifferentiated neoplasia	1 (0.8)
**Monthly family income in US$**	
< 1 MW**	44 (32.8)
1 MW–2 MW	53 (39.5)
> 2 MW	23 (17.2)
No information	14 (10.4)
**Mean (±SD)**	359.80 (±324.31)
**Cervical cancer screening in the previous two years**	
Yes	59 (44.0)
No	75 (56.0)

Abbreviations: FIGO, International Federation of Gynecology and Obstetrics; MW, minimum wage; SD, standard deviation.

** MW, minimum wage (US$ 244.41).

A total of 102 patients required hospital admission (76.1%). The median hospitalization was 7 days (IQR: 3–10), and a total of 10,944 working days were lost due to illness, to treatment, or to death (mean 81.7 ± 76.8). There were 44 surgical procedures performed in 34 patients. Only 6 (13.7%) procedures had curative intent. The most common surgical requirement was abdominal emergencies, and colostomy was necessary in 34.1% of the cases (Table 2).

**Table 2:** Surgical procedures performed on women treated for invasive cervical cancer at the Instituto de Medicina Integral Prof. Fernando Figueira

Procedure	*n* (%)
Abdominal emergency	24 (54.6)
Thoracic procedures*	7 (15.9)
Modified radical hysterectomy with pelvic lymphadenectomy	5 (11.4)
Pelvic exenteration	1 (2.3)
Urinary deviation	3 (6.8)
Others	4 (9.1)

* Tracheostomy (*n* = 3), pleurectomy decortication (*n* = 2), and thoracotomy tubes (*n* = 2).

A total of 102 (76.1%) were treated with chemotherapy, and 89 (66.4%) required radiotherapy. From a societal perspective, the total cost was US$ 644,461.66. This total represents an annual per patient cost of US$ 2,219.73 (or I$ 3,591.72). If the calculated loss of production based on data provided in the interviews were used instead of the Human Capital approach, the total cost would have been US$ 584,048.99, and it would represent an annual cost of US$ 2,011.65 per patient (Table 3).

**Table 3:** Total cost of invasive cervical cancer treatment by costing approach

Cost category	Costs		
	Top-down and human capital US$ (%)	Top-down and interview US$ (%)	Bottom-up and interview US$ (%)
**Direct medical cost**	523,218.22 (81.2)	523,218.22 (89.6)	581,965.75 (90.5)
**Direct nonmedical cost**	22,455.60 (3.5)	22,455.60 (3.8)	22,455.60 (3.5)
**Indirect cost**	98,787.84 (15.3)	38,375.17 (6.6)	38,375.17 (6.0)
**Total cost**	644,461.66(100.0)	584,048.99 (100.0)	642,796.52 (100.0)
**Annual cost per patient**	2,219.73	2,011.65	2,213.99

From the perspective of the patients, there were irrelevant direct medical costs, but patients had direct nonmedical out-of-pocket expenses, even though these costs represented only 3.5% of the total cost. These costs were related mainly to transportation (US$ 91.71 per patient) and food (US$ 67.44 per patient). Accommodation cost only US$ 8.43 per patient. Considering the perspective of the payer, radiotherapy was the most expensive strategy, responsible for 38.2% of the total cost, followed by outpatient chemotherapy (27.4%). From the perspective of the institution, the largest share of resources was used for hospitalization (41.7%), followed by outpatient chemotherapy (31.3%) and radiotherapy (16.6%). Direct medical costs related to surgical procedures were not counted from the perspective of the institution (Table 4).

**Table 4:** Direct medical costs from different perspectives in US$ and mean comparison

Cost of Items	Direct medical costs				
	Perspective of the payer Costs in US$ (mean)	%	Perspective of the institution Costs in US$ (mean)	%	*p-value* *
Hospitalization	116,009.49 (1,197.32)	22.2	242,776.25 (1,811.76)	41.7	< 0.0001
Radiotherapy	199,794.32 (1,491.00)	38.2	96,503.26 (720.17)	16.6	< 0.0001
Outpatient chemotherapy	143,268.17 (1,069.16)	27.4	182,401.07 (1,361.20)	31.3	0.0002
Imaging studies	13,191.16 (98.44)	2.5	53,761.52 (401.21)	9.2	< 0.0001
Laboratorial tests&	6,523.65	1.2	6,523.65	1.1	−
Surgery	44,431.43	8.5	NA		
**Total**	523,218.22 (3,904.61)	100.0	581,965.75# (4,343,03)		0.0237

Abbreviation: NA, not available.

* *t* test (between the cost mean);

& *t* test not performed because those are prices applied by an external laboratory;

# excluded surgery

As can be seen in Table 5, from the perspective of the institution, the staff wages (health and administrative) were responsible for the largest share of the total cost, representing 58.1% of the total.

**Table 5:** Distribution of expenditures from the perspective of the institution, in US$

Cost of Items	Perspective of the institution	%
Staff wages (health and administrative)	338,354.89	58.1
Overheads	139,613.58	24.0
Medications and supplies	103,997.28	17.9
**Total**	**581,965.75**	**100.0**

A year of ICC treatment for the Brazilian population covered only by the SUS (75% of the total population), considering the base case, would represent an estimated financial burden of US$ 25,954,195.04 to the SUS budget.

## Discussion

In our study, we found an annual cost of US$ 2,219.73 (or I$ 3,591.72) per ICC patient from a societal perspective. Applying this annual per patient cost to the estimated incidence of ICC in Brazil in 2014,^3^ the total annual cost of treating ICC in the SUS population would be US$ 25,954,195.04. These results were lower than those reported in another study that estimated the costs of preventing and treating cervical cancer in Brazil using a gross costing methodology. This other study found a total annual cost of US$ 36,448,391.83 for clinical treatment, and of US$ 7,650,810.48 for surgical treatment at the SUS in 2006 US$.^5^ Other previously published studies have reported different annual per patient costs for treating ICC in Brazil, ranging from US$ 3,170.85^26^ in 2006 to US$ 17,517.70^27^ in 2008. In addition, these results were lower than the mean global ICC treatment costs of I$ 16,390.15 described previously.^28^ This discrepancy may be due to differences in costing methodologies, differences in time references, or to the great economic disparities between the regions in Brazil and abroad.^29^

Radiotherapy expenditures accounted for the largest share of total costs (38.2%), followed by outpatient chemotherapy (27.4%). The present study did not include the costs of radiotherapy equipment, since they were purchased > 10 years ago. Nowadays in Brazil, there is a deficit of 255 radiotherapy services, and an investment plan is underway to expand access to radiotherapy.^30^ About US$ 155 million were invested in the acquisition of new equipment between 2017 and 2018, which, if accounted for, would further increase the share of the cost of this treatment.^31^

As new drugs in cancer care are often expensive and are not available through the SUS, many patients turn to the courts to try to receive these new drugs. In 2016, US$ 3,167,701.86 were spent on using the legal process to receive new cancer drugs in Brazil.^32^ It is important to determine where the resources to cover the costs of these medications obtained through the courts will come from, and, therefore, authorities in Brazil have been trying to reach an agreement. The present study found that the SUS spent US$ 523,218.22 for treating and following 134 patients with ICC. From the perspective of the IMIP, the total treatment cost created a budget deficit, and bringing this extra cost to all institutions that treat cancer is unlikely to be sustainable.

The economic burden of cancer care is high worldwide, but it is not equally distributed across all nations. Despite the fact that low- and middle-income countries represent 84.5% of the world population and 61.3% of new cancer cases globally, these areas account for only 6.2% of the financial expenditures on cancer.^33^ The transferability of the findings of economic studies between different settings is widely discussed, and the results of cost evaluations might vary from place to place due to differences in the severity of the disease, to the availability of health care resources, to clinical practice patterns, and to prices.^34^ Whenever possible, each jurisdiction should conduct its own economic studies.^16^

The present study has limitations. First, direct nonmedical costs were obtained via interviews, and no proof of expense was requested. However, direct nonmedical costs represented only 3.5% of the total costs, and variations in them would hardly change the final conclusions. Second, the extrapolation of data from one institution to the entire population of the country leads to approximate results. To accurately measure the cost of ICC treatment for the Brazilian population only covered by the SUS, a very precise costing study would need to include all of the institutions that treat cancer in the country in the microcosting approach, which would probably be unfeasible. Nevertheless, considering that treatment strategies and reimbursements are comparable across institutions for the SUS, the cost of ICC treatment would be expected to be similar across these institutions.

Even though our study is subject to underestimation, it reveals the treatment costs of ICC in a poor region of Brazil where there is still a high incidence of advanced cervical cancer. Screening and treatment of precancerous lesions has been insufficient to reduce the ICC incidence to levels found in developed nations, and we hope that vaccination against HPV will succeed.

In an ideal setting, preventive measures would be fully implemented, and patients who nevertheless developed ICC would be treated with the best evidence-based medicine. In a real scenario, where resources are limited and the known preventive measurements are not yet well implemented, great caution should be exercised in diverting resources to expensive palliative treatments.

## Conclusion

The present study is a detailed analysis of ICC treatment costs that revealed a high financial burden to the health care system in a poor region of Brazil. We have used a microcosting approach and a societal perspective to perform a comprehensive evaluation. Our estimates could be applicable to further evaluations of the cost-effectiveness of preventing and treating ICC and could become a tool for decision-makers in budget planning.
